# The intestine and the microbiota in maternal glucose homeostasis during pregnancy

**DOI:** 10.1530/JOE-21-0354

**Published:** 2022-01-31

**Authors:** Erica Yeo, Patricia L Brubaker, Deborah M Sloboda

**Affiliations:** 1 Department of Biochemistry and Biomedical Sciences, McMaster University, Hamilton, ON, Canada; 2 Farncombe Family Digestive Health Research Institute, McMaster University, Hamilton, ON, Canada; 3 Department of Physiology, University of Toronto, Toronto, ON, Canada; 4 Department of Medicine, University of Toronto, Toronto, ON, Canada; 5 Department of Obstetrics, Gynecology and Pediatrics, McMaster University, Hamilton, ON, Canada

**Keywords:** gestation, insulin secretion, insulin resistance, GLP-1, microbial metabolites

## Abstract

It is now well established that, beyond its role in nutrient processing and absorption, the intestine and its accompanying gut microbiome constitute a major site of immunological and endocrine regulation that mediates whole-body metabolism. Despite the growing field of host-microbe research, few studies explore what mechanisms govern this relationship in the context of pregnancy. During pregnancy, significant maternal metabolic adaptations are made to accommodate the additional energy demands of the developing fetus and to prevent adverse pregnancy outcomes. Recent data suggest that the maternal gut microbiota may play a role in these adaptations, but changes to maternal gut physiology and the underlying intestinal mechanisms remain unclear. In this review, we discuss selective aspects of intestinal physiology including the role of the incretin hormone, glucagon-like peptide 1 (GLP-1), and the role of the maternal gut microbiome in the maternal metabolic adaptations to pregnancy. Specifically, we discuss how bacterial components and metabolites could mediate the effects of the microbiota on host physiology, including nutrient absorption and GLP-1 secretion and action, and whether these mechanisms may change maternal insulin sensitivity and secretion during pregnancy. Finally, we discuss how these pathways could be altered in disease states during pregnancy including maternal obesity and diabetes.

## Introduction

**Figure 1:**
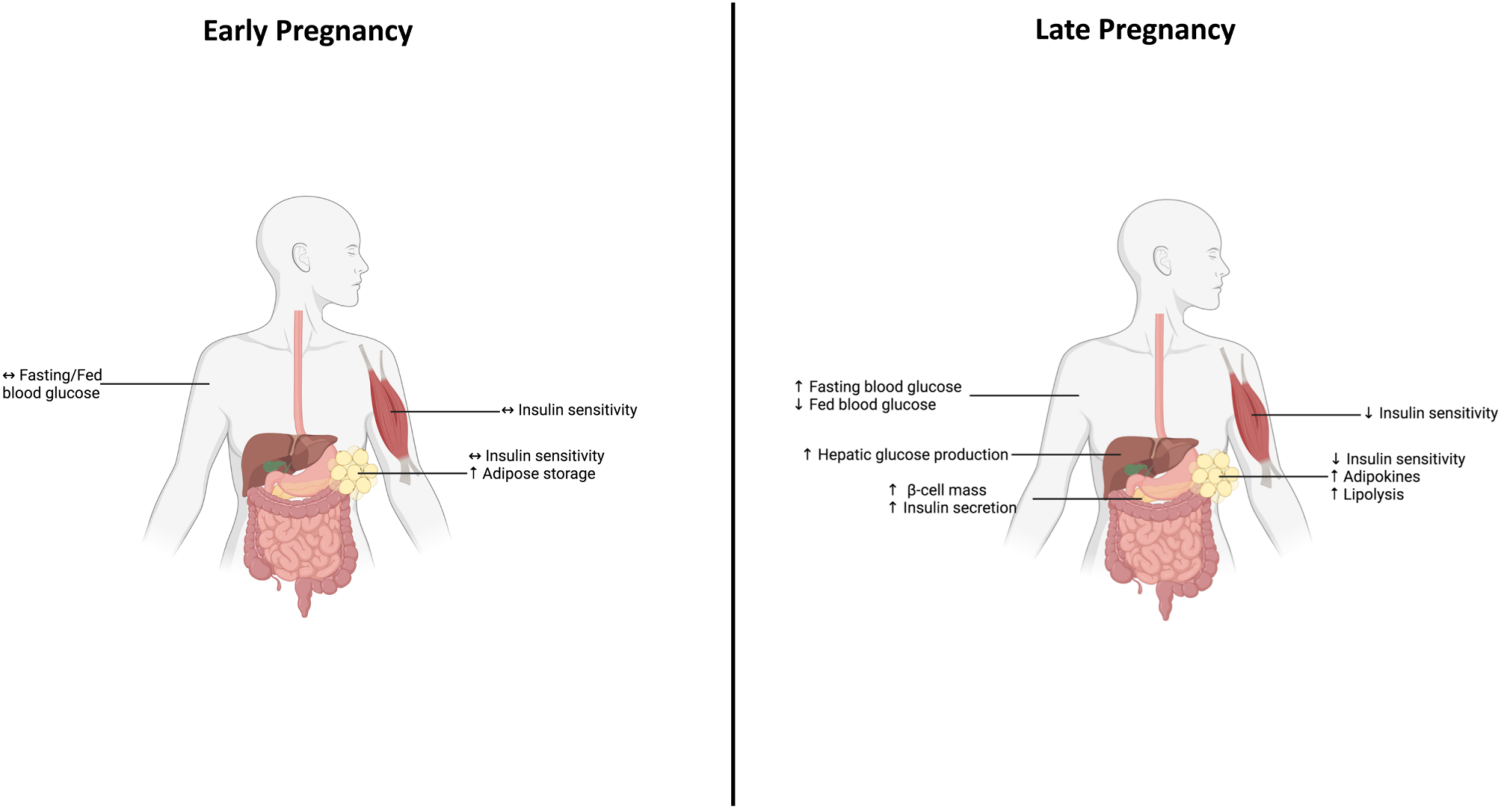
Summary of key changes to maternal glucose metabolism during pregnancy. Diagram highlighting the key maternal metabolic adaptations to pregnancy occurring in early and late pregnancy. Maternal metabolic adaptations occur in order to support increased energy demands during pregnancy. In early pregnancy, this includes increased adipocyte hypertrophy as a form of energy storage for later pregnancy and changes to insulin sensitivity. Later in pregnancy, insulin sensitivity is decreased in peripheral tissues (muscle and adipose) and centrally in the liver and is compensated for by increased insulin secretion from the maternal pancreatic β-cell. Hepatic glucose output is increased, and blood glucose levels are increased in later pregnancy. Together these changes allow for increased glucose transfer to the fetus to support growth.

To accommodate the dynamic energy demands of pregnancy, while still maintaining metabolic homeostasis, significant alterations to maternal metabolism are required (Fig. 1). Impaired or inappropriate maternal adaptations can disrupt the proper division of nutrients between mother and fetus, affecting the health of both (Sferruzzi-Perri *et al.* 2020). Although the mechanisms behind the metabolic adaptations to pregnancy are not fully understood, they are driven, at least in part, by endocrine-mediated pathways (Napso *et al.* 2018). However, it has been suggested that these changes alone are unable to fully explain the maternal metabolic shifts that occur from early to late pregnancy (Kirwan *et al.* 2002).

Early in pregnancy, maternal metabolism is anabolic to prepare for the increased energy demands of fetal and placental growth later in pregnancy. In late pregnancy, maternal metabolism becomes catabolic, and the energy usage shifts from glucose to primarily lipid oxidation, leaving high levels of circulating blood glucose to support placental and fetal growth and metabolism. These shifts in energy usage are mediated by alterations in maternal insulin secretion and sensitivity that occur throughout pregnancy (Lain & Catalano 2007). Until recently, the mechanistic drivers behind these metabolic adaptations were thought to be entirely dependent upon endocrine-related pathways. However, recent discoveries that host-microbe relationships govern glucose regulation in the non-pregnant state, have opened the door to new investigations of the maternal intestine and its bacterial content, as well as to its contribution to metabolic function (Koren *et al.* 2012). To date, the role of the maternal intestine in glucose control has been largely overlooked in the context of pregnancy. This review describes the changes that occur in maternal glucose homeostasis during pregnancy and discusses novel pathways involving the maternal intestine that may regulate these metabolic adaptations.

## Maternal glucose metabolism

It is well established that maternal insulin sensitivity decreases over the course of gestation to facilitate reduced peripheral glucose uptake and increased hepatic glucose production, fueling the increased demand for glucose supply to the fetus (Lain & Catalano 2007). The exact timing of the onset of insulin resistance remains controversial. Both human and rodent studies have described increases (Flint *et al.* 1979), decreases (Catalano *et al.* 1991, Beamish *et al.* 2017)) or no changes (Lind *et al.* 1973) in insulin sensitivity in early pregnancy. In contrast, most data point to a significant reduction in insulin sensitivity in late pregnancy compared to the non-pregnant state (Catalano *et al.* 1991, Musial *et al.* 2016, Beamish *et al.* 2017). Of note, pregnancy-induced hyperglycemia and hyperinsulinemia are not pathological; rather, elevated levels of maternal blood glucose remain within a healthy range due to both the insulin-independent transplacental transfer of glucose to the fetus as well as increased maternal insulin production to compensate for the rising maternal insulin resistance.

So how does the maternal pancreas cope with the sustained insulin demand of pregnancy? Pancreatic β-cells are plastic and can respond to changes in insulin demand and, although this is pathological in metabolic diseases, this same expansion of β-cells occurs physiologically in pregnancy (Butler *et al.* 2010, Salazar-Petres & Sferruzzi-Perri 2021). Under non-pregnant circumstances, β-cell mass is maintained by a balance between increased cell number (proliferation and neogenesis) and cell loss (apoptosis). Over the course of pregnancy, there is an increase in β-cell area (Butler *et al.* 2010) primarily driven by an increase in the number of β-cell clusters, which suggests that neogenesis is a driving force of expansion rather than proliferation (Butler *et al.* 2010). However, the timing and magnitude of this expansion is unclear, and investigations are limited by the scarcity of human pregnant pancreatic samples.

In rodent studies, proliferation is the driving force of β-cell expansion, and the highest levels of proliferation are observed from embryonic days 10–17 (term 20–22 days) (Parsons *et al.* 1992, Beamish *et al.* 2017). More recent studies have identified a population of β-cell progenitors (Ins^+^Glut2^LO^) that are increased prior to pregnancy-related β-cell expansion, although it is unclear how much these cells contribute to expansion (Beamish *et al.* 2017). In addition to β-cell expansion, insulin secretory capacity is also increased in pregnancy. Pancreatic islets isolated from pregnant rodents are more sensitive to and secrete more insulin in response to glucose compared to non-pregnant animals (Green & Taylor 1972). Ultimately, both expansion and increased secretory capacity of β-cells increase insulin production in the pancreas and compensate for the decreased insulin sensitivity in the mother.

These highly coordinated adaptations in insulin sensitivity and secretion ultimately allow the transfer of maternal glucose to the fetus (Fig. 1). These adaptations, however, must be finely tuned, as excessive insulin resistance and/or failure to increase β-cell mass can result in pregnancy complications including gestational diabetes mellitus (GDM, diabetes first diagnosed in pregnancy, and resolving after pregnancy) (Buchanan 2001). (Fig. 1). While maternal adaptations to support fetal growth in pregnancy have been well studied for over 20 years, our understanding of the mechanisms that drive these adaptations is still not fully understood.

## Our current understanding of the regulation of maternal glucose metabolism during pregnancy

**Figure 2:**
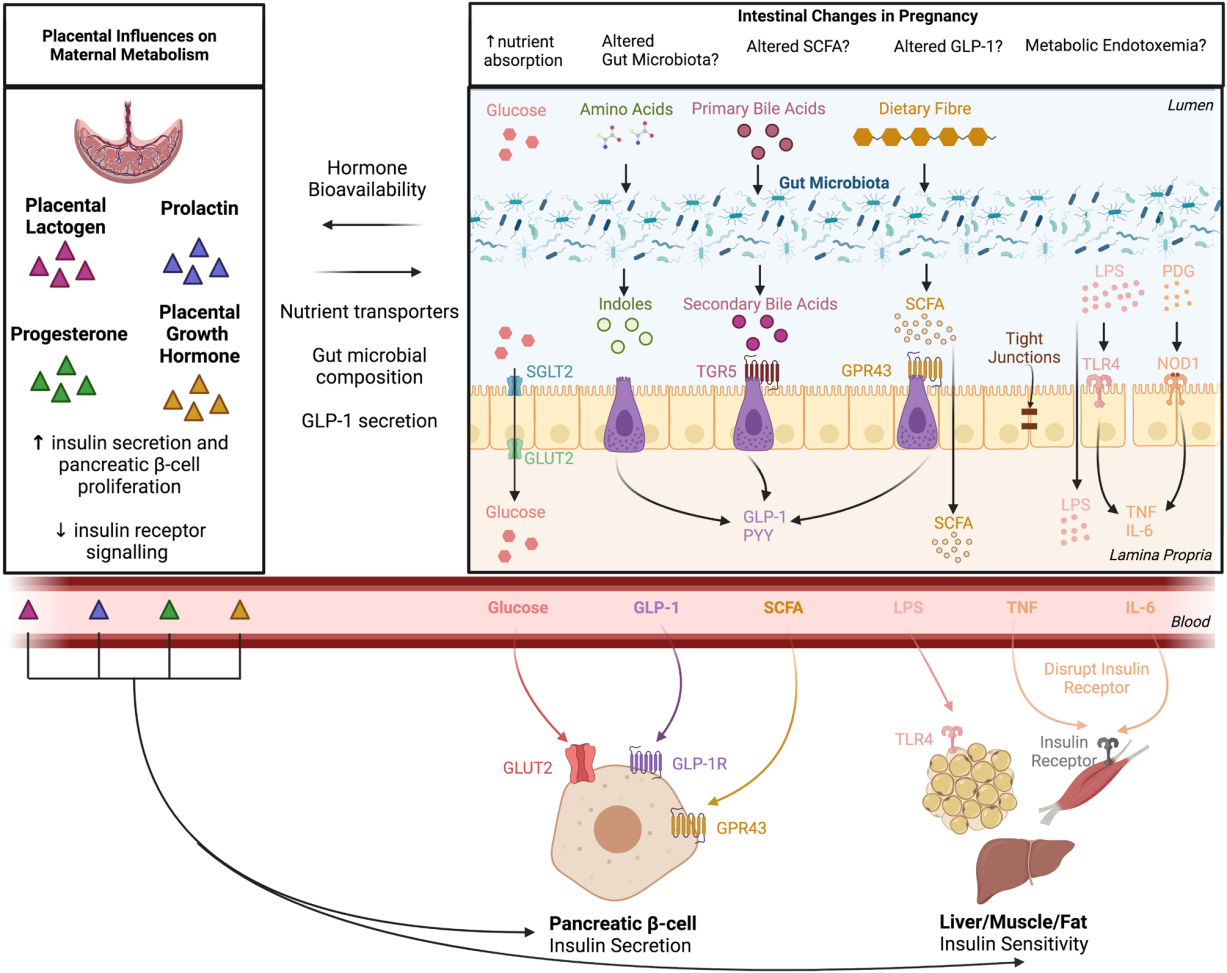
Summary of proposed intestinal influences on maternal metabolism. Schematic diagram highlighting the various intestinal pathways that may impact maternal metabolic adaptations to pregnancy. This includes interaction with placental derived hormones, changes to intestinal nutrient transport, gut microbial composition, changes to microbially derived metabolites (i.e. SCFAs, Indoles, BAs), gut hormone secretion and bacterial components activating inflammatory pathways.

A number of placental hormones contribute to the changes in maternal insulin sensitivity and secretion, as reviewed elsewhere (Napso *et al.* 2018). Briefly, placental hormones, including placental lactogen (PL), placental growth hormone (PGH) and progesterone, disrupt insulin receptor signaling (Beck & Daughaday 1967, Wada *et al.* 2010) and, thus, result in a progressive state of maternal insulin resistance which drives maternal β-cells to synthesize and secrete more insulin. This expansion of insulin-producing β-cells coincides with the onset of PL, PGH and prolactin (PrL) secretion from the placenta. As PL, PGH and PrL induce β-cell proliferation and also increase glucose-stimulated insulin secretion (Brelje *et al.* 1993, Huang *et al.* 2009), these hormones therefore regulate both insulin sensitivity and secretion (Fig. 2). However, while the mechanisms underlying the endocrine regulation of insulin receptor signaling and insulin secretion are clear, circulating levels of PL and PrL do not correlate with decreased maternal insulin sensitivity among healthy pregnant individuals, suggesting the influence of other factors (Kirwan *et al.* 2002).

Pregnancy is characterized not only by changes in endocrine hormones but also in the immune system. Inflammatory factors secreted by maternal immune cells and adipose tissue, such as tumour necrosis factor (TNF) and interleukin (IL)-6, are associated with insulin resistance during pregnancy (Kirwan *et al.* 2002, Todoric *et al.* 2013). Plasma TNF levels are positively correlated with insulin resistance in early and late pregnancy, although the source of this TNF remains unclear. However, in the context of obesity-related insulin resistance, increased production of TNF and other inflammatory cytokines from excess adipose tissue impairs insulin receptor signaling by promoting serine rather than tyrosine phosphorylation of the downstream mediator, insulin receptor substrate 1, via induction of stress kinase (JNK or IKK/NFkB) pathways (Tanti & Jager 2009). It seems likely that increased TNF levels may act similarly during pregnancy, but in a non-pathological manner due to the concomitant, physiological upregulation of insulin production.

## Putative intestinal factors regulating maternal glucose metabolism

Recent clinical and experimental evidence has demonstrated that both the intestinal tract and the gut microbiota play key roles in metabolic homeostasis in the non-pregnant state. Although poorly explored in the context of pregnancy, it is likely that the changes that are known to occur in intestinal function and microbiota composition during pregnancy may also contribute to the maternal metabolic responses to pregnancy.

During pregnancy, increased food intake (hyperphagia) is accompanied by structural and functional changes to the gastrointestinal tract (Peña-Villalobos *et al.* 2018). Given that collection of gastrointestinal tissue from pregnant individuals is often not feasible, much of what is known about gut physiology during pregnancy has been obtained from studies on rodent and sheep models. In rats, intestinal length and weight increase during pregnancy and peak by late lactation (Cripps & Williams 1975, Burdett & Reek 1979, Johnson *et al.* 2019). Similarly, pregnant ewes have 45% heavier small intestines compared to non-pregnant controls (Fell *et al.* 1972). The mucosal epithelium, the main site of nutrient absorption, also increases in weight by 30–40% in rats ((Burdett & Reek 1979), commensurate with increased small intestinal villus length at mid-to-late pregnancy compared to early pregnancy (Sabet Sarvestani *et al.* 2015). Together, these adaptations increase the surface area available for nutrient absorption. Although few studies to date have specifically measured nutrient transport across the intestine during pregnancy, increased expression of the intestinal GLUT5 and SGLT1 glucose transporters has been reported in pregnant rats (Teerapornpuntakit *et al.* 2014).

Whether pregnancy-related hormones govern intestinal adaptations to pregnancy is unclear. Few studies have investigated the mechanisms underpinning these intestinal adaptations. One study in non-pregnant rats treated with PrL demonstrated increased glucose transport across the intestinal epithelium (Mainoya 1975), but this stands in contrast to another study that showed decreased expression of glucose transporters in response to PrL administration (Charoenphandhu *et al.* 2008). Some studies have shown that nutrient absorption is influenced by a reduction in gastrointestinal transit that occurs in both human and rodent pregnancy, thereby increasing the time available for absorption of dietary nutrients (Wald *et al.* 1981, Liu *et al.* 2002). Although the pregnancy-related hormones progesterone and oxytocin have been reported to increase gastrointestinal transit time in male rats (Liu *et al.* 2002), whether increases in nutrient absorption support the early anabolic phase of pregnancy metabolism and/or the increasing energy demands of late pregnancy (Fig. 2) is still unknown.

### Gut hormones and their impacts on metabolism

The intestine is the largest endocrine organ system in the body, with at least 15 different cell types, each with a unique hormone secretion profile. Many of these hormones play roles in the regulation of intestinal digestion, absorption and motility (Murphy & Bloom 2006). Some of the key hormones include serotonin (involved in gut motility, glucose metabolism) (Kim *et al.* 2010, Martin *et al.* 2019), peptide YY (involved in appetite suppression) (Batterham & Bloom 2003), and cholecystokinin (involved in pancreatic enzyme secretion, appetite regulation and gut motility) (Gibbs *et al.* 1997, Suzuki *et al.* 2010). However, of all the potential endocrine factors that could contribute to maternal metabolic adaptations to pregnancy, glucagon-like peptide-1 (GLP-1) is particularly interesting because of its relationship with the gut microbiota, intestinal structure and function, and pancreatic insulin synthesis and secretion. Its actions on pancreatic β-cells, in particular, make it a compelling putative factor linking pregnancy-associated changes in gut microbiota, pancreatic β-cell expansion and maternal glucose regulation.

### Glucagon-like peptide 1

GLP-1 has been well studied in its role in maintaining glucose homeostasis, and its physiology and clinical applications in non-pregnant subjects have been reviewed extensively elsewhere (Müller *et al.* 2019). GLP-1 is an incretin; stimulating glucose-dependent insulin secretion, as well as increasing insulin biosynthesis and, at least in rodents, inducing β-cell proliferation. GLP-1 also acts as an indirect stimulator of insulin sensitivity through its effects on satiety which leads to weight loss. Studies in subjects with type 2 diabetes mellitus (T2DM) who have impaired insulin secretion suggest that decreased GLP-1 secretion and/or action may play a role in diabetes pathogenesis. As such, GLP-1 receptor agonists and GLP-1 degradation inhibitors are used therapeutically to improve insulin secretion in individuals with T2DM (Meier 2012). There is some evidence that these therapeutics may be effective in pregnant individuals with T2DM or GDM; however, they are not currently recommended due to possible harmful effects on the developing fetus (Chen *et al.* 2020). Moreover, it is unclear whether such changes in GLP-1 contribute to the changes in maternal insulin secretion and β-cell expansion that occur during pregnancy (see more below).

GLP-1 is a product of the proglucagon gene (*Gcg*), expressed in intestinal L-cells and in α-cells of pancreatic islets as well as neurons in the caudal brainstem and hypothalamus (Müller *et al.* 2019). Transcription factors PAX6, CDX2 and CREB regulate the expression of *Gcg*in L- and α-cells (Jin 2008). Wnt signaling molecules, GSK-3B and TCF4, are also involved in regulating transcription of the *Gcg* gene (Yi *et al.* 2005, 2008), and these same Wnt molecules drive the development of the intestinal L-cell through gradients in intestinal stem cells during differentiation. To our knowledge, no studies have investigated these signaling pathways in the maternal L-cell during pregnancy, although genetic variants of TCF proteins (nuclear Wnt pathway components) have been associated with the development of GDM (Shaat *et al.* 2007, Reyes-López *et al.* 2014). But whether Wnt signaling impairs intestinal secretion of GLP-1 during pregnancies complicated by GDM is unknown.

Expression of *Gcg* is regulated not only at the transcriptional level but also post-translationally. Once expressed, GCG undergoes tissue-specific cleavage by the prohormone convertase (PC) enzymes (PC1/3, PC2). PC2 acts primarily in α-cells to generate glucagon, whereas PC1/3 is expressed in the L-cell and the nervous system. In the gut, PC1/3 cleavage of GCG liberates GLP-1 in two bioactive isoforms: GLP-1(7-36)NH_2_, which makes up the majority of the secreted GLP-1 and, to a lesser extent, the equipotent GLP-1(7-37)NH_2_ (Orskov *et al.* 1993, Dhanvantari *et al.* 1996). GLP-1 is secreted by the L-cell in response to nutrient stimuli from the gut lumen, although L-cells also respond to gut microbial components and metabolites, as discussed below (Petersen *et al.* 2014, Beumer *et al.* 2018, Lund *et al.* 2020).

L-cells are also responsive to insulin (Yi *et al.* 2008, Lim *et al.* 2009); these findings led to the notion of a feed-forward loop between the L-cell and the β-cell that is regulated by luminal nutrients and blood glucose levels, supporting a potential role for GLP-1 as a key factor in maternal metabolic adaptations to pregnancy. GLP-1 secretion is impaired in obesity and T2DM, and whether insulin resistance of the L-cell may explain impaired insulin secretion in these diseases (Verdich *et al.* 2001) is unclear; but, given the insulin-resistant state of pregnancy, it is possible that maternal L-cells may be less responsive to insulin in pregnancy. This has yet to be investigated.

#### GLP-1 during pregnancy

Despite the significant changes in insulin secretion and sensitivity that occur during pregnancy, little is known of the potential role played by GLP-1 in maternal glucose metabolism. Some data exist to suggest that GLP-1 levels rise with advancing gestation (Valsamakis *et al.* 2010). Experimental work in mice shows an increase in the number of GLP-1 producing L-cells in the maternal intestine at term pregnancy (Moffett *et al.* 2014), and the absence of all GCG-derived peptides (in *Gcg* ^−/−^ mice) during pregnancy results in decreased maternal insulin levels (Sugiyama *et al.* 2012). GLP-1 receptor knockout mice are unable to increase β-cell mass during pregnancy compared to WT controls (Moffett *et al.* 2014) suggesting that, indeed, there may exist a pregnancy-induced change in GLP-1 regulation of pancreatic insulin secretion.

Despite this potential role, no studies have investigated the drivers of increased GLP-1 activity during pregnancy. Studies in non-pregnant mice have identified a stimulatory role for progesterone receptor signaling in GLP-1 secretion (Flock *et al.* 2013, Zhang *et al.* 2014) suggesting that progesterone could elicit GLP-1 secretion, but this has not been investigated in humans. One human study reports that individuals diagnosed with polycystic ovarian syndrome (PCOS) (and consequently altered sex hormone levels) have blunted GLP-1 levels in response to a meal – an effect that is associated with glucose intolerance (Svendsen *et al.* 2009, Aydin *et al.* 2014). Whether estradiol, testosterone, other sex hormones, or indeed any of the pregnancy-related hormones directly target intestinal L-cell GLP-1 synthesis and secretion in humans is not well established. Even less is understood about how these endocrine stimulators may facilitate GLP-1 induced changes in the pancreatic β-cell during pregnancy.

Several studies exist to suggest that GLP-1 plays no role in pregnancy-related changes in insulin secretion, reporting both no changes in fasting plasma GLP-1 levels (Bonde *et al.* 2013) and a diminished GLP-1 response to glucose during the third-trimester compared to post-partum (where post-partum measures were a proxy for the ‘non-pregnant’) (Hornnes *et al.* 1981). However, many limitations confound investigations of GLP-1 and pregnancy. Data are limited by measuring GLP-1 in the circulation, which does not reflect L-cell secretion of GLP-1. This is important because GLP-1 can act locally, within the gut, to stimulate the gut–pancreatic–neural axis (Waget *et al.* 2011). Richer data sets are needed, with larger samples sizes, and more controlled experimental designs that include both pregnant and true non-pregnant subjects, rather than post-partum proxy measures, as the post-partum period is another time of great change in maternal metabolism (Stuebe & Rich-Edwards 2009).

Furthermore, while the vast majority of GLP-1 is produced by L-cells in the intestine, moderate increases in intra-islet GLP-1 production in the pancreas have been reported in the context of increased metabolic demand such as in the prediabetic and diabetic states, as well as during pregnancy (Kilimnik *et al.* 2010, Moffett *et al.* 2014). This intra-islet GLP-1 likely acts in an autocrine/paracrine manner and therefore would not affect circulating GLP-1 levels (Panaro *et al.* 2020). Nevertheless, one must consider not only when, but also where samples are collected and what they represent. Indeed, once secreted, GLP-1 has a very short half-life in circulation and is rapidly degraded by dipeptidyl peptidase IV (DPP IV, a serine protease), into the inactive forms GLP-1(9-36)NH_2_ and GLP-1(9-37)NH_2_ (Kieffer *et al.* 1995). To our knowledge, DPP IV levels have not been measured in the maternal circulation during pregnancy. DPP IV has, however, been detected in syncytiotrophoblast-derived extracellular vesicles (STB-EV) which are released from the placenta into the maternal circulation. These DPP IV-positive STB-EVs are increased in pregnancies complicated by GDM (Kandzija *et al.* 2019), suggesting that the metabolic challenge of gestational diabetes affects the circulating levels of GLP-1. Nonetheless, despite gaps in our understanding of the mechanism(s), it appears likely that GLP-1 presents a novel factor that contributes to pregnancy-induced changes in maternal glucose metabolism. Future studies investigating GLP-1 action on insulin secretion in pregnancy should consider the dynamics of secretion and degradation in determining GLP-1 levels, as well as the resultant activity of the GLP-1 receptor in the main target, the maternal pancreatic β-cell.

### Microbial impacts on host metabolism

Intestinal nutrient digestion and absorption are assisted by a community of microorganisms present in the intestine that ferment otherwise non-digestible dietary components (Oliphant & Allen-Vercoe 2019). Consisting of trillions of microbes that have a mutualistic relationship with the host, these organisms are collectively referred to as the microbiota. It has long been recognized that the gut microbiota and their genetic and metabolic material – the microbiome – regulate a wide array of physiological systems (Rajilić-Stojanović & de Vos 2014). Recent studies have only just begun to uncover the relationships between these communities and human health and disease and, in particular, their roles in nervous and immunological development and function, as well as metabolism (Tremaroli & Bäckhed 2012, Burcelin 2016, Dinan & Cryan 2017).

Investigation of the relationship between gut microbiota and host metabolic development and function has been facilitated by the use of germ-free (GF) mice that lack all microbial communities. The absence of the gut microbiota in these mice results in a leaner phenotype (Backhed *et al.* 2004), in association with a decreased ability to absorb energy from the gut (Turnbaugh *et al.* 2006), and decreased fat storage (Backhed *et al.* 2004). When challenged with a high-fat diet, GF mice also have greater insulin sensitivity and glucose clearance compared to conventionally-raised, specific pathogen-free (SPF) mice (Rabot *et al.* 2010). Furthermore, when GF mice are colonized with microbiota from SPF mice, these new ‘conventionalized-GF’ mice display increased adiposity and insulin secretion (Backhed *et al.* 2004), further indicating that, indeed, the gut microbiota contribute to host metabolism and may be associated with metabolic dysfunction.

In humans, a number of metabolic disorders including obesity and T2DM have been associated with an altered or ‘dysbiotic’ gut microbial composition (Ley *et al.* 2006, Larsen *et al.* 2010, Wu *et al.* 2017). In particular, decreased alpha (within sample) and beta (between samples) bacterial diversity appear associated with metabolic impairments (Ley *et al.* 2005).

#### Pregnancy-related changes in gut microbes and their function

In light of known associations between host metabolism and gut microbial composition in the non-pregnant state, a number of studies have also examined these relationships in pregnancy. Reports that the composition of the gut microbiota changes over the course of pregnancy exist, but these require confirmation (Table 1). In their seminal study, Koren* et al*. demonstrated decreased alpha diversity (which has been previously associated with metabolic impairments) in third-trimester maternal fecal samples compared with the first trimester; additionally, the relative abundance of Proteobacteria and Actinobacteria phyla was increased and the* Faecalibacterium* genus was decreased in the third-trimester (Koren *et al.* 2012). Higher Proteobacteria and decreased *Faecalibacterium* have been associated with a number of metabolic and inflammatory disorders due to a reduction in the production of beneficial metabolites (see below) (Qin *et al.* 2012, Chassaing *et al.* 2015). In a separate study, *Clostridium histolyticum*, *Akkermansia muciniphila* and *Bifidobacterium*were increased in third-trimester samples (Collado *et al.* 2008, Nuriel-Ohayon *et al.* 2019). Changes in the composition of the gut microbiota over the course of pregnancy have also been reported in mouse models, notably increases in the *Akkermansia, Clostridium*, *Bacteroides* and *Bifidobacterium*genera have been observed not only at the onset of pregnancy but throughout the course of gestation (Gohir *et al.* 2019, Nuriel-Ohayon *et al.* 2019). Decreased abundance of the Firmicutes and Tenericutes phyla have also been reported (Gohir *et al.* 2019). While it is difficult to associate changes to specific taxa with function due to the redundancy of the gut microbiota, there are a number of established metrics of ‘dysbiotic’ or ‘unhealthy’ microbiota, such as the ratio of Bacteroidetes to Firmicutes (positive correlation with glucose tolerance) (Ley *et al.* 2005). Interestingly, the changes in these taxa are similar to those seen in non-pregnant individuals with obesity and T2DM and may, therefore, represent a similar microbial phenotype whether physiologically during pregnancy or in disease states (Pitlik & Koren 2017).

**Table 1:** Summary of changes to the gut microbial composition during health pregnancy.

	Mouse			Human						Primate
	Gohir et al. 2015	Elderman et al. 2018		Collado *et al.* 2008	Koren *et al.* 2012	Yang *et al.* 2020	Jost et al.2014	DiGiulio *et al.* 2015	Qin *et al.* 2021	Mallott *et al.* 2020
Location				Finland	Finland	China	Switzerland	United States	China	
Sample population	C57BL/6J mice *n* = 5 non-pregnant*n* = 5 pregnant	C57BL/6J mice *n* = 10 non-pregnant*n* = 10 pregnant	BALB/c *n* = 10 non-pregnant*n* = 10 pregnant	Normal weight *n*= 36Over weight *n*= 18	*n*= 91 pregnant women of varying BMI, GDM status.	*n*= 1479 healthy pregnant individuls	*n* = 7 healthy pregnant individuals	*n* = 40 healthy pregnant individuals	*n* = 47 healthy pregnant individuals	*n* = 15 Phayre's Leaf Monkeys
Study design	Longitudinal	Cross-sectional	Cross-sectional	Longitudinal	Longitudinal	Cross-sectional	Longitudinal	Longitudinal	Longitudinal	Cross-sectional
Time of sampling	Gestational days 0.5, 6.5, 10.5, 14.5, 17.5	Pregnant mice at E18.5	Pregnant mice at E18.5	T1 (10–15 weeks) T3 (30–35 weeks)	T1 (13.84 weeks) T3 (33.72 weeks)	Various gestational ages	Prepartum (3–7 weeks) Postpartum (3–6, 9–14, 25–30 days)	Weekly	T1 (11–13 weeks) T2 (23–28 weeks) T3 (33–38 weeks)	Non-pregnant Pregnancy Lactation
Detection Methods	16s rRNA sequences (V3) Illumina	Mouse Intestinal Tract Chip (MITChip)		FCM-FISH qPCR	16s rRNA sequences (V1/V2) 16 Illumina	s rRNA sequences (V4) Illumina	16s rRNA qPCR	16s rRNA sequences (V4/V5) Illumina	16s rRNA sequences Illumina	16s rRNA sequences (V4/V5) Illumina
Alpha Diversity	–	↓ (shannon)	↓ (shannon)	–	↓ (Faith's PD)	–	–	–	–	↓(Shannon)
Observed taxa^a^										
Actinobacteria	↑	–	–	–	↑	–	–		–	–
Proteobacteria	–	––	–	–	↑	–	–	–	–	–
Enterobacteriaceae	–	–	–	–	↓	–	–	–	–	–
Lachnospiraceae	–	–	–	–	↑	↑	–	-–	–	–
Ruminococcaceae	–	–	↑	–	↑	↓	–	–	–	–
Akkermansia	↓	–	–	↑	–	↑	–	–	–	–
Allobaculum	↑	–	↑	–	–	–	–	–	–	–
Anaerotruncus	↓	–	↑	–	–	–	–		–	–
Bacteroides	↑	–	↑	↑	–	↑	–	–	–	–
Bifidobacterium	↑	–	–	↑	–	↑	–	–	–	–
Clostridium	↑	–	–	↑	–	–	–	–	–	–
Collinsella	↑	–	↑	–	–	↑	–	–	–	–
Coprobacillus	↓	–	↑	–	–	–	–	–	–	–
Escherichia	↑	–	↑	–	–	–	–	–	–	–
Eubacterium	–	–	↑	–	↑	–	–	–	–	–
Faecalibacterium	–	–	↑	–	↑	↓	–	–	–	
Lactobacilus	–	–	↑	–	↓	–	–	–	–	–
Lactococcus	↑	–	↑	–	–	–	–	–	–	–
Prevotella	↑	–	↑	–	–		–	–	–	–
Propionibacterium	–	–	↑	–	↓	–	–	–	–	–
Roseburia	↑	–	↑	–	–		–	–	–	–
Staphylococcus	–	–	↑	↑	–	↑	–	–	–	–
Streptococcus	–	–	↑	–	↓	–		–	–	–
Subdoligranulum	–	–	↑	–	↑	–	–	–	–	–

^a^Taxa summarized in the table reflect taxa which were observed to be altered in >1 study.

But while these studies have observed clear shifts in microbial composition with advancing gestation, others have found only weak correlations (Table 1). Such studies showed that fecal microbiota in individuals over the course of pregnancy and post-partum showed no changes to overall community structures (DiGiulio *et al.* 2015) but demonstrated alterations in the abundance of specific taxa (Yang *et al.* 2020, Qin *et al.* 2021). Differences in the participant population, the timing of sampling, the sequencing methods, and analyses likely all contribute to the variable results, since established protocols are only just emerging in perinatal microbial analyses (Theis *et al.* 2019, Kennedy *et al.* 2021). Robust and in-depth investigations are required to determine whether gestational age-related changes in maternal intestinal microbial composition actually occur. If changes in composition do occur over the course of pregnancy, further investigation into whether these result in altered function is also required. Indeed, Yang* et al*. recently found that, despite seeing significant relationships between the abundance of specific taxa and gestational age, the microbial composition was more strongly associated with individual characteristics (i.e. BMI, gestational weight gain, pregnancy-related diseases) than with gestational age (Yang *et al.* 2020). Whether individual host characteristics drive microbial composition and/or changes to composition functionally contribute to host physiology in pregnancy is unclear; however, it is likely that both variables will have a significant impact (Kovacs *et al.* 2011, Jena *et al.* 2017).

While the mechanisms underpinning changes in gut microbial composition during pregnancy remain unclear and, indeed, whether functional outputs of these shifts are meaningful, some experimental data suggest that sex hormones may be formidable contributors to microbiota variability. In mice, males and females have distinct gut microbiota after the onset of puberty (Markle *et al.* 2013, Steegenga *et al.* 2014, Kaliannan *et al.* 2018). Since the levels of both estrogen and progesterone are greatly increased during pregnancy, it is likely that these hormones also influence pregnancy gut microbial composition. Indeed, these hormones have been associated with changes in diversity and the growth of specific taxa (in particular, species of the *Bifidobacterium*and *Bacteroides*genera) in both non-pregnant and pregnant models (Kornman & Loesche 1982, Nuriel-Ohayon *et al.* 2019, Mallott *et al.* 2020). Conditions of hormonal imbalance, including PCOS or primary ovarian insufficiency, have been linked to alterations in gut microbiota and are associated with metabolic impairments (Tremellen & Pearce 2012, Wu *et al.* 2021). Furthermore, the gut microbiota is altered in non-pregnant mice that lack estrogen receptor β compared to WT mice (Menon *et al.* 2013). This host endocrine-microbe relationship may also be bidirectional; inactive estrogen metabolites that are excreted in the bile can be reactivated by gut bacteria, which can then be reabsorbed into circulation, affecting their bioavailability (Flores *et al.* 2012). This may represent a unique pathway by which the intestine plays a role in maternal metabolic adaptations to pregnancy, through alterations in the microbiota that affect the bioavailability of estrogens. Endocrine control of gut microbial composition is thus likely to play a role in pregnancy, given the dynamic changes that occur in hormone levels and may, therefore, have effects on overall metabolism both directly (as mentioned previously in this review) and indirectly through the gut microbiota (Fig. 2).

#### Bacterial metabolites as drivers of metabolic change

The first evidence that the maternal gut microbiota may directly contribute to maternal metabolic adaptations was obtained in GF mice that were colonized with the microbiota from third-trimester pregnant individuals. These mice had increased body fat deposition and decreased insulin sensitivity compared to mice colonized with first trimester microbiota (Koren *et al.* 2012). This is consistent with data in mice colonized with feces from obese and diabetic individuals (Ridaura *et al.* 2013). How this occurs is unclear but likely occurs either through bacterial components, bacterial products or bacterial metabolites.

Gut microbiota exert many of their effects on the host via bacterial components (Kikuchi *et al.* 2018, Solito *et al.* 2021). The paracellular movement of live bacteria and/or their components through an impaired intestinal barrier has been suggested to contribute to T2DM- and obesity-related insulin resistance; a state referred to as metabolic endotoxemia (Cani *et al.* 2007). This process is facilitated by components of the bacterial membrane, including lipopolysaccharides (LPS) and peptidoglycan, that bind to innate immune sensors, toll-like receptor 4 (TLR4) and nucleotide-binding oligomerization domain-containing protein 1, respectively, resulting in disruption of insulin receptor signaling. These sensors also stimulate the production of inflammatory cytokines such as TNF and IL-6 that, in turn, can also impair insulin receptor signaling (Schertzer *et al.* 2011). Although it is unclear whether the changes in insulin action during pregnancy are facilitated by similar mechanisms, increased gut permeability has been observed during pregnancy (Gohir *et al.* 2019) (Fig. 2) and, therefore, this pathway could also operate during pregnancy.

Bacteria also produce metabolites that are beneficial to health (Fig. 2), including the fermentation of non-digestible carbohydrates into short-chain fatty acids (SCFA), the processing of bile acids (BA) into secondary BAs, and the unique processing of amino acids such as tryptophan into indole compounds. The most well studied are bacterial SCFAs; SCFAs can be used directly by the host for energy, act as substrates for gluconeogenesis and lipogenesis, and bind receptors present in numerous organ systems (Tan *et al.* 2014). Activation of SCFA receptors (GPR42, GPR43 and GPR109a) has been implicated in many metabolic processes including energy harvest, adipose and skeletal muscle function, and insulin sensitivity and secretion, as well as gut hormone production including GLP-1 (Koh *et al.* 2016). In rodents, the administration of SCFA (in particular, butyrate) prevents diet-induced obesity and the development of diabetes by improving glucose control and insulin sensitivity (Gao *et al.* 2009). Long-term dietary interventions in humans are inconclusive, but some have demonstrated insulin-sensitizing effects of SCFA (Bouter *et al.* 2018).

Although still a developing field, studies have begun investigating the role of SCFAs in maternal metabolism during pregnancy. In mice, the concentration of SCFAs in cecal contents and plasma are not different between non-pregnant and pregnant mice, but the ratios of different SCFAs are significantly altered, with higher proportions of acetate and propionate, and a lower proportion of lactate, in pregnant mice (Fuller *et al.* 2015). Increased propionate is consistent with observed increases in gluconeogenesis during pregnancy, as propionate is known to be gluconeogenic (den Besten *et al.* 2013). Altered SCFA proportions can also affect receptor activation. The SCFA receptors, GPR41 and GPR43, are expressed on L-cells on both the luminal and basolateral membranes, and GLP-1 secretion is stimulated by acetate, propionate and butyrate (Lin *et al.* 2012, Tolhurst *et al.* 2012, Yadav *et al.* 2013). This presents an interesting hypothesis that microbial metabolite-induced changes in gut hormone production could influence insulin secretion during pregnancy. Indeed, studies in pregnant individuals and their newborns have shown negative correlations between serum propionate and acetate levels with maternal weight gain, maternal glucose and hormone levels (including insulin), and neonatal weight (Priyadarshini *et al.* 2014, Szczuko *et al.* 2020), potentially implicating SCFA-induced changes in host metabolic function perhaps through GLP-1. When the SCFA receptor *Ffar2 (*GPR43) is knocked out, pregnant mice are unable to produce adequate levels of insulin (Fuller *et al.* 2015). Whether this was due to changes in GLP-1 was not investigated but, in non-pregnant obese mice, it has been proposed that SCFA-mediated GLP-1 secretion may be involved in the insulin-sensitizing and glucose-lowering effects of butyrate (Yadav *et al.* 2013).

Insulin sensitivity and secretion are also influenced by the bacterial metabolism of bile acids, although this relationship is less studied than that of SCFAs. Primary BAs are produced and secreted by the liver into the upper intestine and can be reabsorbed (acting as a negative feedback loop on BA production), or deconjugated by microbial enzymes into secondary BA(Ahmad & Haeusler 2019). BA receptors (FXR and TGR5) are present in the liver, intestine and islets and are able to regulate glucose metabolism by altering glucose absorption in the gut and increasing insulin secretion from the β-cells (Ahmad & Haeusler 2019). The FXR receptor has a higher affinity for primary BA, while TGR5 has a higher affinity for secondary BA; thus, the composition of BA both in the intestine and in the circulation can greatly affect receptor activation and downstream activity (Kawamata *et al.* 2003, Kalaany & Mangelsdorf 2006). In the context of obesity and T2DM, BA composition is altered and has been suggested to contribute to insulin resistance (Haeusler *et al.* 2013). Secondary BA receptors are present on intestinal enteroendocrine cells where they promote the secretion of intestinal hormones (Tolhurst *et al.* 2012, Brighton *et al.* 2015); indeed, BAs are well-known stimulants of GLP-1 through TGR5 receptors on L-cells (Katsuma *et al.* 2005, Brighton *et al.* 2015). Increased serum BAs have been observed during pregnancy, as well as increased microbial deconjugation of primary BAs to secondary BAs, resulting in a higher proportion of secondary BAs (Ovadia *et al.* 2019). No studies to date have investigated whether increased maternal secondary BA levels are associated with changes in GLP-1. Alternatively, increased secondary BAs could alter maternal glucose metabolism directly at the level of the pancreas, activating TGR5 receptors on the β-cell to increase insulin secretion during pregnancy, although this has not been investigated.

In addition to SCFAs and BAs, other bacterial components and products modulate L-cell function and GLP-1 secretion. The major component of the outer membrane of Gram-negative bacteria, LPS, stimulates the expression of both *Gcg* and *Pcsk1* (which encodes for the prohormone processing enzyme PC1/3) as well as GLP-1 secretion, in a TLR4-dependent manner, both *in vitro*and *in vivo* in rodents and humans (Nguyen *et al.* 2014, Lebrun *et al.* 2017). In contrast, chronic exposure to the proinflammatory cytokine TNF has been found to suppress both *Gcg* expression and GLP-1 secretion (Gagnon *et al.* 2015). Other less studied microbial metabolites have also been demonstrated to stimulate GLP-1 production including indoles, a product of tryptophan metabolism (Chimerel *et al.* 2014); a bacterial cytolytic peptide Hld (Tomaro-Duchesneau *et al.* 2020); and other specific bacterial components that act in a similar manner (Yoon *et al.* 2021). Given the wide-reaching effects of bacterial components, products and metabolites it is clear that a number of bacterial-associated factors may be key effectors of gut microbial influence on host metabolism. Whether these factors act similarly in the context of maternal metabolic adaptations that occur during pregnancy is, as of yet, completely unknown and warrants further investigation.

## Clinical relevance

Because intestinal adaptations during pregnancy are likely necessary for appropriate maternal metabolic function, the intestine may provide not only a point of intervention but may also be a source of biomarkers when considering diagnosis, prevention and treatment of poor maternal and fetal outcomes. Although a number of risk factors are used clinically to assist in identifying individuals at risk of pregnancy-associated metabolic complications, specific biomarkers that can parse out individuals that need increased support vs specific pharmacological intervention strategies, are critical in improving maternal and fetal health and well-being.

In Canada, about one-third of individuals entering pregnancy are overweight or obese (Dzakpasu *et al.* 2014), with 16.9% of live births being complicated by maternal hyperglycemia worldwide (https://www.diabetesatlas.org). Obesity is characterized as a BMI greater than 30 kg/m^2^ with systemic low-grade inflammation and is often accompanied by metabolic dysfunction and insulin resistance (Després & Lemieux 2006). The change in insulin sensitivity that occurs from pre-gravid to early pregnancy is inversely correlated to maternal weight and pre-gravid insulin sensitivity, suggesting an impaired ability to metabolically adapt to pregnancy in obese women, and increasing their risk for GDM (Catalano & Ehrenberg 2006). As a result, the physiological decrease in insulin sensitivity that normally occurs during pregnancy acts as a catalyst, putting obese individuals at higher risk of developing GDM. Maternal obesity, with or without GDM, is associated with poor maternal metabolic adaptations, due to impaired pre-gravid insulin sensitivity and/or impaired insulin secretion during pregnancy (Alvarado *et al.* 2021). Furthermore, GDM is associated with an increased risk to the mother of developing T2DM later in life, as well as increased risk of metabolic disease in the offspring, heightening the need to manage GDM (Damm *et al.* 2016). While GDM is used as a clinical diagnosis, it has been proposed that maternal glucose control should be viewed as a continuous spectrum, whereby the degree of glucose intolerance is also related to negative outcomes (Hyperglycemia and Adverse Pregnancy Outcome studies (Scholtens *et al.* 2019)). The extent to which the maternal intestine regulates poor maternal metabolic adaptation under these circumstances is unclear; however, this notion is gaining interest, given the increasing evidence of the involvement of the gut in metabolic disorders in the non-pregnant state.

Obesity and T2DM have been associated with dysbiotic gut microbiota in non-pregnant individuals and, more recently, in cases of maternal obesity and GDM. Several studies have identified a gut microbial signature associated with an obese pregnancy, in mouse models (Vrieze *et al.* 2014, Gohir *et al.* 2019, Wallace *et al.* 2019) (Table 2). Given that obesity is a risk factor for GDM, many of the same signatures also appear to be associated with GDM, although it is difficult to completely separate the two conditions, as many individuals who develop GDM are also overweight or obese. Lower microbial richness and diversity are observed in individuals with, or who later develop, GDM compared to normoglycemic women (Kuang *et al.* 2017, Cortez *et al.* 2019, Ma *et al.* 2020), although these results are confounded by high BMI in individuals with GDM. Fecal microbiota from overweight and obese pregnant women in their third-trimester demonstrate an increased abundance of *Staphylococcus aureus*compared to lean pregnant women (Collado *et al.* 2008). Studies in mice have also shown increased *Akkermansia muciniphila* and decreased *Ruminoccaceae* in fecal microbiota from pregnant mice fed a high-fat diet (Gohir *et al.* 2019, Wallace *et al.* 2019). Several independent studies also found that genera belonging to the family Ruminococcaceae were significantly decreased in overweight and obese individuals who eventually developed GDM (Crusell *et al.* 2018, Ma *et al.* 2020). Ruminocaccaceae were also negatively correlated with maternal fasting blood glucose measures, although these changes were no longer observed when the data were adjusted for BMI (Ma *et al.* 2020). Ruminococcaceae species are known to be SCFA producers and have also been associated with improved carbohydrate metabolism and lower long-term weight gain (Menni *et al.* 2017). An interesting study by Ye* et al*. further demonstrated that individuals with GDM could be categorized into those in whom lifestyle modifications were able to control glycemia, vs those in whom lifestyle modifications were ineffective based solely on their gut microbiota (Ye *et al.* 2019). These data highlight the complexity of glucose control and risk factors for GDM such as obesity, with the emerging data suggesting that the gut microbiota likely play a role; whether this is a direct or indirect role is unknown.

**Table 2:** Summary of changes to gut microbial composition in gestational diabetes mellitus.

	Human								
	Kuang *et al.* 2017	Mokkala et al. 2017	Crusell *et al.* 2018	Cortez *et al.* 2019	Ye *et al.* 2019	Ma *et al.* 2020	Wu et al. 2020	Xu et al. 2020	Chen et al. 2021
Location	China	Finland	Denmark	Brazil	China	China	China	China	China
Sample population	43 GDM+ 81 GDM- GDM- group older, higher BMI	15 GDM+ 60 GDM- overweight/obese	50 GDM+ 161 GDM- overweight/obese	26 GDM+ 42 GDM- GDM group older and higher BMI	36 GDM+ 16 GDM-	98 GDM+ 98 GDM- GDM gorup higher BMI	23 GDM+ 26 GDM-	30 GDM+. 31 GDM-	30 GDM+ 28 GDM-
Study design	Cross-sectional	Cross-sectional	Cross-sectional	Cross-sectional	Cross-sectional	Cross-sectional	Cross-sectional	Cross-sectional	Cross-sectional
Time of sampling	T2 (26 weeks)	T1 (12 weeks)	T2 (28 weeks)	T3 (~33 weeks)	T2 (24–28 weeks)	T1 (10–15 weeks)	T3	T3 (~38 weeks)	T3 (24–28 weeks)
Detection methods	Metagenomic Sequncing Illumina	16s rRNA sequencing Illumina	16s rRNA sequencing (V1/V2) Illumina	16s rRNA sequencing (V4) Illumina	16s rRNA sequencing (V3/V4) Illumina	16s rRNA sequencing (V4) Illumina	Metagenomic Sequncing Illumina	16s rRNA sequencing (V3/V4) Illumina	16s rRNA DNA Microarray
Alpha diversity	↓ (Shannon, Observed)	–	–	↓ (Chao1)	–	↓ (various)	–	–	–
Taxa altered in GDM^a^									
Lachnospiraceae	–	–	–	↑		↑	–	–	–
Ruminococcaceae	–	↑	↑	↑		↓	–		–
Alistipes	↓	–	–	–		–	↓	↓	–
Bacteroides	↑	–	↓	–		–	↑	–	–
Blautia	–	–	↑	↑		–	–	–	–
Clostridiales	↓	↓	–	–		–	–	–	–
Clostridium	↑	–	↓	–		–	–	–	–
Colinsella	–	–	–	↑		–	–	–	↓
Eubacterium	↓	–	–	–		↓	–	–	–
Faecalibacterium	–	–	↑	–		–	–	–	–
Fusobacterium	↓	–	–	–		–	–	–	–
Haemophilus	↓	–	–	–		–	–	↑	↑
Lactobacillus	↑	–	–	–		–	↓	–	–
Parabacteroides	↑	–	–	–		↓	–	–	–
Phascolarctobacterium	↑	–	–	–		–	–	↓	–
Roseburia	↓	–	–	↑		–	–	–	–
Ruminoccocus	–	–	↑	↑		–	–	–	–
Ruminoclostridium	↓	–	–	–		–	–	–	↑

^a^Taxa summarized in the table reflect taxa which were observed to be altered in >1 study.

While alterations in the abundance of specific taxa may provide us with an idea of what a ‘healthy’ gut microbiota during pregnancy looks like, it is unclear whether changes to the gut microbial composition are causal or are a symptom of the disease. To our knowledge, only one study has demonstrated that the composition of the microbiota can recapitulate phenotypes associated with GDM, wherein GF mice colonized with microbiota from a pregnant person diagnosed with GDM were found to be hyperglycemic, compared to those colonized with microbiota from a normoglycemic pregnant individual (Liu *et al.* 2020). Taken together, these data suggest that the microbial composition is altered in pregnancies that are complicated by metabolic disorders and may contribute to disease pathogenesis; however, the mechanisms that drive this relationship remain unknown. Given our knowledge of the beneficial effects of SCFAs in contexts outside of pregnancy, a reduction in SCFA producers (such as Ruminococcaceae species) may be one mechanism by which the microbiota participates in GDM pathogenesis, and could be an avenue of study and, potentially, intervention. Furthermore, while much of the host-microbe literature considers a ‘dysbiotic’ microbiota to be disease associated, changes to microbial communities in pregnancy may be a healthy adaptation that is necessary for maternal adaptations. A longitudinal study of gut microbial changes during healthy pregnancies and those complicated by obesity/GDM is necessary to understand how the maternal microbiota changes over time and how it responds to different metabolic pressures.

While there is currently limited evidence describing mechanisms that underpin the gut microbial influence on GDM pathogenesis, some studies have begun to investigate whether microbial interventions can be used to prevent or treat GDM. Despite the many clinical trials that have probed the use of probiotics for a variety of different disease states, there are few data to suggest a mechanism of action and most studies have been predominantly speculative. Indeed, to our knowledge, the largest trial addressing this is the Study of Probiotics in Gestation (SPRING) trial, which included 411 overweight or obese pregnant individuals and concluded that probiotics were not effective in preventing GDM (Callaway *et al.* 2019). Notwithstanding, the use of probiotics is growing widely in popularity, including before, during and after pregnancy, although their effectiveness in preventing negative perinatal outcomes is unclear (Jarde *et al.* 2018, Amirpour *et al.* 2020, Lopez-Tello *et al.* 2021). Furthermore, caution is warranted; a recent Cochrane systematic review suggests that probiotics used for the treatment of GDM may increase the risk of pre‐eclampsia compared to placebo (RR 1.85) and may also increase the risk of hypertensive disorders of pregnancy (RR 1.39) (Davidson *et al.* 2021). Pre-eclampsia is also associated with an altered gut microbiota (Miao *et al.* 2021). Nonetheless, the authors concluded, given the risk of harm and little observed benefit, that current trials proceed with caution.

The ultimate goal of GDM treatment is to manage hyperglycemia in order to protect both the mother and the fetus from adverse outcomes later in life. While it is unclear whether any microbial interventions will do this, incretin-based therapies are extensively used for the treatment of hyperglycemic disorders such as T2DM but may not be safe or effective for use in pregnancy. Little is known of the role of GLP-1 in increasing insulin secretion in a healthy pregnancy, and even less so in pregnancies complicated by obesity and GDM. Fasting and glucose-stimulated levels of GLP-1 increased over the course of pregnancy in normoglycemic women; however, women with GDM had decreased levels of GLP-1 throughout gestation (Lencioni *et al.* 2011, Sukumar *et al.* 2018, Mosavat *et al.* 2020). Meanwhile, others have suggested that GLP-1 plays little role in GDM pathogenesis (Hornnes *et al.* 1981, Cypryk *et al.* 2007). Since a link between SCFAs, BAs and GLP-1 secretion exists, microbial interventions may very well also act to improve GLP-1 levels in GDM, although this has not been investigated.

## Conclusions and future directions

Evidence from studies investigating metabolic dysfunction in the non-pregnant state has provided a wealth of knowledge about the role of the intestine in whole-body metabolism. However, there is a lack of data describing these pathways during pregnancy. The intestine is a new avenue of investigation to understand how maternal metabolic adaptations are initiated and regulated. As described in this review, a number of studies have emerged investigating changes in gut microbiota during pregnancy. Future studies should shift their focus from identifying changes in gut microbial composition during pregnancy to understanding whether these compositional changes have functional impacts on maternal metabolism. Specifically, do the gut microbiota contribute to normal pregnancy-related metabolism and/or do alterations in gut microbial composition during pregnancy contribute to pregnancy-related complications? Further, it is known that inadequate maternal metabolic adaptations can result in pregnancy complications including GDM. The current standard of care for individuals with GDM or T2DM during pregnancy includes lifestyle interventions as well as insulin or metformin therapy (American Diabetes Association 2020). However, insulin and metformin are unable to mitigate the negative effects of GDM on the offspring (Barbour *et al.* 2018, Hanem *et al.* 2019, Zhu *et al.* 2019). The growing popularity of gut health has accelerated the use of probiotics and other microbial interventions, including during pregnancy, although our limited understanding of the intestine and the gut microbiota during pregnancy prevents us from knowing whether these interventions are safe and effective. It is also unclear whether GLP-1-related interventions would be effective in GDM as our understanding of the role of GLP-1 and DPP IV during pregnancy is limited. Additional studies are needed to measure GLP-1 activity during pregnancy taking into account DPPIV-mediated degradation and intra-islet secretion.

Further studies of intestinal mechanisms regulating pregnancy-associated changes in maternal insulin sensitivity and secretion are required, in order for us to make well-informed, evidence-based decisions to intervene and mitigate the impacts of maternal disease states, such as obesity and GDM, on glucose metabolism.

## Declaration of interest

The authors declare that there is no conflict of interest that could be perceived as prejudicing the impartiality of this review.

## Funding

E Y is supported Farncombe Family Digestive Health Research http://dx.doi.org/10.13039/100005622 Institute Student Fellowship and Ontario Graduate Fellowship. P L B is supported by a Tier 1 Canada Research Chair in Vascular and Metabolic Biology. D M S is supported by a Tier 2 Canada Research Chair in Perinatal Programming.

## Author contribution statement

E Y wrote the manuscript and compiled the figures. P L B and D M S edited and wrote the manuscript. All authors have reviewed and approved this manuscript.
